# Supplementary material for: Molecular and Functional Characterization of Elovl4 Genes in Sparus aurata and Solea senegalensis Pointing to a Critical Role in Very Long-Chain (>C24) Fatty Acid Synthesis during Early Neural Development of Fish
Source: Int J Mol Sci. 2020 May 15;21(10):3514. doi: 10.3390/ijms21103514 (PMC7278935; doi:10.3390/ijms21103514)
Supplement: Supplementary file 1 [file ijms-21-03514-s001.pdf]

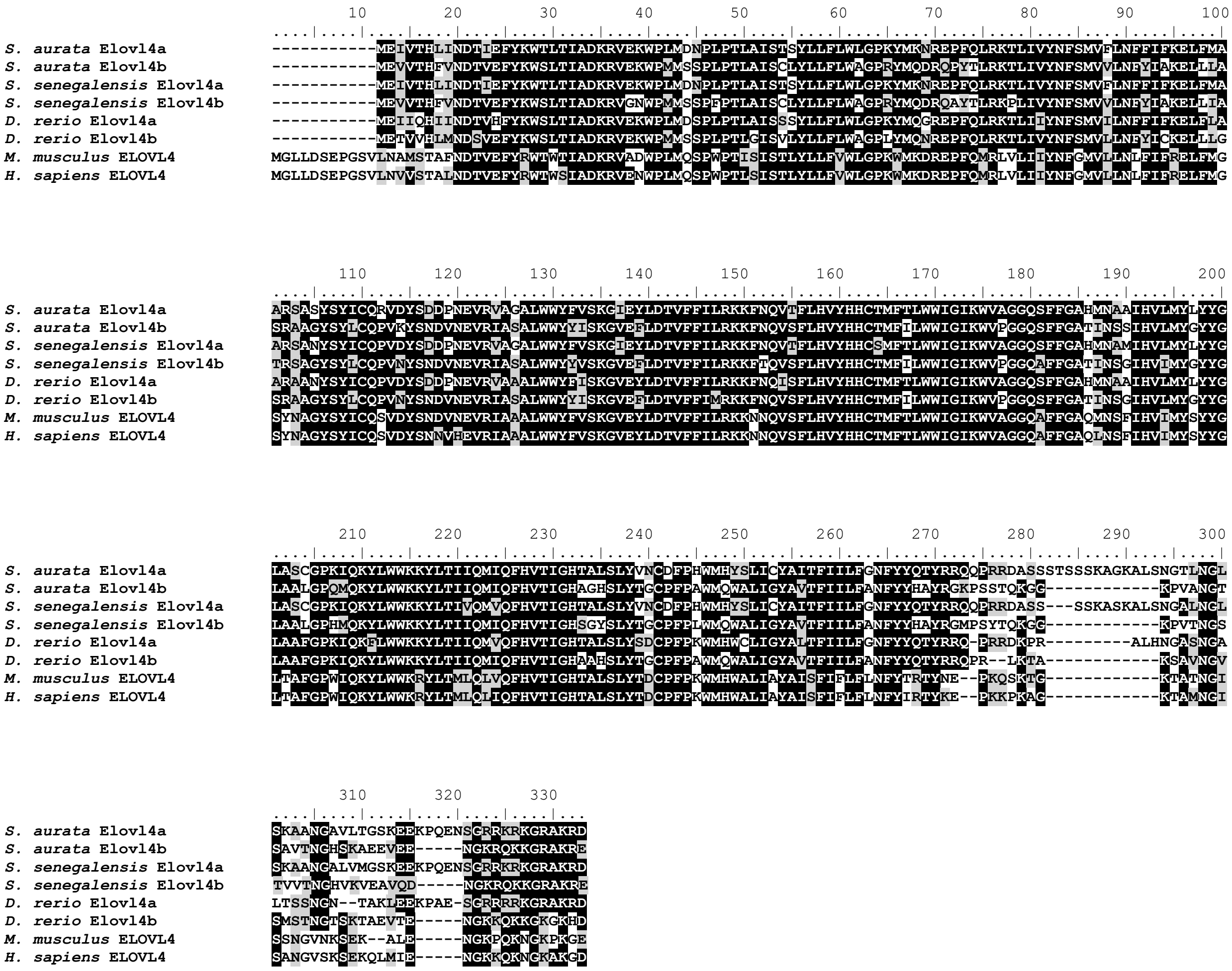

Supplementary Figure S1. ClustalW amino acid alignment of the deduced *Sparus aurata* Elovl4a (gb|QES86604.1), *Solea senegalensis* Elovl4a (gb|QGA31141.1), *Sparus aurata* Elovl4b (gb| QES86605.1), and *Solea senegalensis* Elovl4b (gb| QGA31140.1). Identical residues are shaded black and similar residues are shaded grey. The four (I-IV) conserved motifs of Elovl enzymes: I (KXXEXXDT), II (QXXFLHXXHH), III (NXXXHXXMYXYY) and IV (TXXQXXQ), as well as the endoplasmic reticulum (ER) retrieval signal (RXKXX) at the C-terminus, are indicated by squares.
